# The Hospital Nursing Supplement

**Published:** 1894-09-01

**Authors:** 


"The Hospital" Nursing Mirror.

Being the Extra Nursing Supplement of "The Hospital" Newspaper.

[Contributions for this Supplement should be addressed to the Editor, The Hospital, 428, Strand, London, W.C., and should have the word "Nursing" plainly written in left-hand top corner of the envelope.]

### News from the Nursing World.

### HOLIDAYS AND NEWS.

At this season of the year, when most people are taking their holiday, it is difficult, if not impossible, to keep in touch with all that is going on in the nursing world. Of course the holidays make a great difference in the work, especially as the hospitals are relatively empty, and the sick relatively few during the summer as compared with the winter months. Still, we think that all our readers might usefully co-operate with us by sending from time to time, to a greater extent than they do at present, items of news. A short account of all promotions, new features, increased comforts, and any changes in system which tend to promote the progress or the happiness of those affected by them. If those of our readers, for example, who are now taking a holiday would visit the nursing or medical institutions which they come across at such times and send us their impressions much good might result. In wishing, therefore, the best of good holidays to those who are fortunate enough to have them, we venture to hope some of them at any rate may take these words to heart, remembering that an editor's work is never done.

### NURSING AT GUY'S.

The salaries of the nursing staff at Guy's Hospital have been entirely rearranged. Formerly the nurse, on completion of her training, only received £20 per annum, beyond which sum there was no definite prospect of increase, though several of the older nurses were in receipt of £25 per annum, in addition to in and out door uniform and free laundry. The new scale for staff nurses provides salaries of £25 for the fourth year, £28 for the fifth year, and £30 for the sixth year of service in the hospital. Beyond this, a nurse is encouraged to join the Royal National Pension Fund, and if she does so at the age of 28, she receives indirectly an increase of £6 9s. a year to her salary. On the other hand, the remuneration of probationers has been diminished, and is at present £8 for the first year, £12 for the second year, and £18 for the third year. The dietary of the nurses has been improved, with the addition of pudding every day for dinner, dessert on Sundays, and a cold meat supper on three, instead of one evening of the week. The new dietary is much appreciated by the nurses; their health during the past year has been exceptionally good; and several changes have been made which have materially increased the comfort of the nursing staff. Miss Nott Bower is credited by the treasurer with having initiated these changes, which have contributed greatly to the health, comfort, and efficiency of the nursing staff. We congratulate Miss Bower and Dr. Perry upon the deserved popularity of Guy's as a training school, a fact which is placed beyond dispute by the circumstance that 2,500 applications were received during the past year from would-be lady pupils and probationers.

### A MODEL TIME TABLE.

It is apparent that the off-duty time of the nurses and probationers at Chelsea Workhouse Infirmary is arranged on a far more liberal scale than would at first be imagined from Miss de Pledge's modest reference to it in her admirable article in the *Westminster Review.* The time table in use at the institution, of which she is the valued Matron, is a model one, and a reproduction of it would have greatly enhanced the interest of her paper. It is specially instructive to note that the plan of letting the day nurses all go off duty at half-past seven p.m. works satisfactorily, and also that every nurse is allowed to go out once on Sundays, and from May to October passes are granted from eight to ten in the evening, in addition to the afternoon leave. In the course of the alternate week, when a half-day of eight hours is given to a nurse, she has also on one or two other afternoons from two to five o'clock free.

### UNDESIRABLE POSTPONEMENTS.

"I never let anything interfere with a nurse's time off duty," remarks a Matron who evidently excels as an organiser and supervisor of nurses; and it is evident that the human machinery works without friction under her management. When a nurse's annual leave is suddenly stopped on the eve of her holiday or a probationer's "two hours' off" curtailed, it is certain that there is "something wrong" in the administration of the institution which thus proves itself without provision for emergencies. The postponement of annual leave is more serious than appears at first sight. It is only due at the expiration of a certain term of service, and it has then become a necessity and not a luxury in the nurse's life, moreover, it is the worker's right, and certainly not a favour to be withheld or granted at the caprice of an official. Nurses' holidays should not be lightly altered when once fixed, for the unselfish women who submit cheerfully to sudden disappointments suffer quite as much inconvenience from disarranged plans and postponed relaxation as any confirmed grumbler who airs loudly her opinion that "it's a horrid shame" to hold over a well-earned holiday.

### STEWARDESSES.

Some years ago The Hospital advocated the employment of trained nurses as stewardesses, and the idea has been satisfactorily adopted. But all candidates for posts on board ship should fully understand that to fill such positions satisfactorily they must be something more than good nurses—they must be good travellers, resourceful, and healthy. The duties are by no means light, and it needs determination and management to get them done with the punctuality demanded on a ship. But this same strict punctuality secures that time off duty is accurately granted, and that meals are not interfered with. An active, healthy woman may lead a very happy, busy life on board ship; but the duties of a stewardess cannot be properly performed by a broken-down or incompetent nurse. To a good nurse who is feeling rather overdone with hospital air, and with many years of ward life, the benefits gained by a sea voyage are many and obvious.

### THE STATE HOSPITALS OF ENGLAND.

Dr. Rhodes, chairman of the Northern Workhouse Nurses' Association, Manchester, declares that the public conscience appears to have been roused, and that there is a strong desire that something should be done to make workhouse infirmaries worthy of the title at the head of this paragraph. He rightly maintains that reform can be best secured by providing trained nurses, thoroughly acquainted with the duties in a workhouse. It has been found by experience that nurses trained in first-class workhouse hospitals like Birmingham, Chorlton, Manchester, and Marylebone are better suited for the work than those obtained from general hospitals. He shows that the greatest impediments to the employment of trained nurses in workhouses are the master and matron, because they are unfortunately too often unconscious of their own ignorance of scientific nursing, and do very much less than they might to make the nurses comfortable. They do these things better in Scotland, where the Board of Supervision require one trained nurse for every twenty cases, and if the workhouse infirmary contains upwards of sixty beds a lady superintendent must be appointed in addition, and in such cases the matron has no authority or jurisdiction, and of course no responsibility. Why is it that the Local Government Board, who are really responsible for the present disgraceful condition of many sick wards in workhouses in England, do not issue a similar order to that in force in Scotland? Surely Sir Walter Foster must be alive to the importance of such a reform, and we look to him, and through him to the President of the Local Government Board and its medical department, to prepare and issue such an order without delay.

### NURSES AND PATIENTS.

The *Daily Telegraph* gives an account of a legacy which has been left to a nurse by a patient, because the doctors told the patient candidly that it was to the nurse rather than to them that she owed her restoration to health. One day recently the patient wrote to one of the missionaries attached to a metropolitan police-court, who had helped the nurse in question to be trained, stating that she wished to make her will in favour of the nurse. The missionary advised a pause for consideration. Was she dealing justly with her relatives? Oh, yes! the relatives were only cousins, and were all richer than she. So the patient made her will, by which she left £6,000 to the nurse, who is a widow with three children, and appointed the missionary executor and trustee, with a small legacy for his trouble. We have always maintained that grateful patients should communicate with the secretary of the Royal National Pension Fund before making a nurse a present, as they could in this way better promote her best interests and welfare. Few patients will ever be able, and fewer still will be willing, to leave a legacy of £6,000 to a nurse, but all well-to-do patients can show their gratitude to a really efficient nurse by paying in a sum of money to the Pension Fund on her behalf, and so directly benefiting the nurse, and so indirectly help to raise the efficiency of private nursing throughout the country.

### OCCASIONAL HOLIDAYS.

The authorities of the Bradford Infirmary have set a good example to English hospital authorities. They have recently granted a whole day's holiday to each member of the nursing staff, the expenses being defrayed by the management. In order to secure the maximum of benefit to nurses, the authorities at Bradford planned out a pleasant excursion for each day, and so ensured the happiness of each of their staff. This idea might be adoped in London in the interests of the patients as well as of the nurses, and we shall hope to hear that some wealthy governors or their wives have planned the excursions and applied to the committees for the necessary leave. A day off duty, when it is spent in relaxation and exercise, must always add to the efficiency of the work done wherever the system prevails. The movement has already spread to Plymouth, where it is proposed to form a committee to raise funds for the relaxation of the nurses at the Fever Hospital, and there is every reason why it should be universally popular in the near future.

### THE NURSE DOLLS.

A party of our nurse dolls are again on their travels, and after appearing at Thame, at an entertainment in aid of the nursing fund, they will proceed to Penrith to assist at a bazaar.

### DISTRICT NURSES IN ADELAIDE.

At a large public meeting held in Adelaide it was recently decided to organise a permanent district nursing society. The work already done by one trained nurse amongst the poor of the district has been eminently successful, and the doctors as well as their patients are most appreciative of the aid received from her. Many serious cases of illness have been attended, and Port Adelaide has appealed for a nurse of its own. The new society hopes in time to provide trained district nurses for every part of the colony, and the primary necessity for a central home was fully acknowledged by the large and representative gathering at the Church Rooms, Adelaide.

### SHORT ITEMS.

Nurse M'Gillivary, of the Leith Links Hospital, has been presented with a photographic album by several of the patients nursed by her during the recent small-pox epidemic, in appreciation of the kindness shown to them while under her care. The presentation was made by Dr. Beveridge.—We regret to notice that although the special committee appointed to consider the provision of better nursing accommodation at the Worcester General Infirmary reported that sketch plans had been prepared for the erection of a nursing home at the estimated cost of £5,000, the Executive Committee has determined to delay the proposal for the present owing to a want of funds.—The congregation of St. John's Church, Burgess Hill, subscribed £14 10s. in aid of the Village Nurses' Fund, and the friendly societies made a collection at their church parade on Sunday last in aid of the same object.—The public ought to make themselves acquainted with the work of the Workhouse Nurses' Association, and of the local associations of the same type, so as to secure that the Local Government Board inquiries, set on foot at their instance, shall receive full sympathy and support. In this way we might speedily obtain many much-needed reforms in the treatment of the sick in workhouses all over the country.

### On General Nursing.

By Rowland Humphreys, M.R.C.S., L.R.C.P.Lond.

### XXVII.—NURSING OF BURNS.

There is no doubt that burns cause more deaths in proportion to their frequency than any other form of injury, and from the fact that women wear more voluminous and more combustible garments, &c., and in the course of their household duties, are more in contact with fire than men, they appear to be the more frequent sufferers, and to be liable to more severe burns. This, of course, omits those employments in which men are engaged in manipulating molten or other heated bodies.

The management varies with the degree of the burn, and with the stage of healing which has been reached.

The degrees of burn have been divided into six, according to the depth to which it has gone, the sixth involving the whole of the limb.

The immediate treatment resolves itself into treatment of the shock, usually very severe, and the relief of pain. These two indications may be met by the use of opium, either hypodermically or by the mouth, and by the protection of the wound from the air. The latter may be carried out by the use of cold water rags, or baths if the surface is extensive; a continuous stream of water passing over the burn is found to be very comforting, and to be of great service, especially if it contains an antiseptic (such as boracic acid) dissolved in it. Greasy applications, such as vaseline and boracic acid and (the abominable) Carron oil, are also in general use. Flour and almost any innocuous powder dusted on the surface is also of value. The application of a weak solution of cocaine to the surface of the wound is of great service where there is severe pain. All blisters should be pricked, and their contents squeezed out and the skin pressed down. Rest to the injured part, as well as to the patient, should be ensured. When the immediate effects have passed off the separation of the slough begins, commencing with inflammation of the surrounding tissues. This does not begin till after the second day.

The inner dressings should not be removed till it becomes absolutely necessary to do so, and the pain thus caused is often so great as to necessitate the administration of chloroform. The sloughs should never be pulled away, both on account of pain and for fear of hæmorrhage from one of the vessels (usually a vein) implicated in the burn. Its removal may be accelerated by the use of some stimulating application, such as Ung. Elemi, or some turpentine liniment. Various applications are in use for preventing the decomposition of the slough, such as iodoform, salol, bismuth subnitrate, and such like. It should be remembered that raw surfaces absorb applications very readily, and that poisoning may result.

As the sloughs separate they should be gradually cut off at their edges, and as soon as the wound is clean skin-grafting should be commenced. The grafts should be minute pieces of skin, cut off so as to avoid bleeding, applied near the edge of the ulcer, and an inch apart, and covered with oiled silk, moistened simply with olive oil. The wound should not be disturbed for two clear days.

One of the great advantages derived from the use of grafts is that skin grows from them, and the scar which necessarily follows the destruction of tissue is much lessened in extent, and when it contracts, as it does sooner or later, the terrible deformities thus produced will be greatly lessened. To assist this object extension of the limb or part affected should be kept up for a considerable time, and especially during the process of healing. This is effected much in the same way as in keeping up extension in a case of hip disease. The limb below the wound is covered by strapping, which is also used to attach a stirrup to its extremity; from this a cord runs over a pulley with a weight attached to its end. If the burn affects the neck or other similarly situated part, an apparatus specially designed for the purpose must be applied so as to prevent the contraction.

As to the complications of burns, shock (killing within the the first day or two), exhaustion, erysipelas, cerebral complications (especially in children), bronchitis, &c., where the chest has been burned, and inflammation of the larynx, where the burn or scald has affected it, have to be guarded against as far as possible.

Shock has already been spoken of; delirium often occurs early, and rigors may come on during the same period. Sleeplessness has to be met, and may be treated either by opium or by sulphonal or chloralamide or paraldehyde. There is a tendency in all cases of burns to ulceration of the intestine, the duodenum, the first part of the intestine, being especially frequently affected. This ulceration, according to the latest investigations, seems to be due to septic influences, and there is too often no warning of its presence till hæmorrhage from mouth or intestine, peritonitis, or the signs of perforation, show its extent. It is therefore necessary to be on the outlook for diarrhœa, abdominal pain, or similar symptoms, and to be very cautious in the use of aperients.

The diet of a burn case will be regulated so that the food may be as completely digested and as nourishing as possible; and as after any injury the digestion, together with the other functions of the body, are more or less in abeyance for some days, it should not be given in a large quantity, but rather often and in small amount. Stimulants may be administered if necessary, but as they tend to increase the fever due to reaction, they should be avoided as much as possible. The danger due to injury to any special organ, such as the eye, must be treated in the manner special to them, the general dangers remaining the same.

### Hospitals in Brazil.

### SANTA CASA DE MISERICORDIA.

Besides the St. Sebastian Hospital, I have only visited one other as yet, namely, the great "Misericordia," or more correctly, "Santa Casa de Misericordia." In England there is no building to compare with it for grandeur, magnificent workmanship, and rich endowment. The wards are splendid, containing 34 beds, floors beautifully polished (not a speck of dust anywhere), all the woodwork oak, or some similar wood. Deep panels all round, above which the walls are tiled. The corridors are very fine indeed, and the bath-rooms are more like old Roman ones, being marble. I shall never forget the appearance of the laboratory and dispensary, worked entirely by the "sisters," and magnificently fitted up, evidently having cost thousands of pounds. We went through the kitchens, also presided over by the sisters. Everything was in the most perfect order, and every utensil as bright as a looking-glass. As we went through the wards the same thing forcibly struck us as at St. Sebastian, namely, the lack of nurses.

We went in uniform (grey and white) and the sisters were most kind to us, and asked us to go and see them often. In the linen-room they always keep 3,000 sets of clothing in stock for the use of patients, for whom there is accommodation for no less than 1,500. We were taken into their chapel—a really lovely place, so exquisitely fitted up.

There are several branches of the Misericordia Hospital, this being the head. There is no great scope for trained private nurses in Rio at present. We have had a few cases, but, as a rule, find people prefer their own "old style" of attendant.

Later on we are going to visit the Berri Hospital, of which I shall hope to send you a description. Cornstalk.

### Holidays in Japan.

(By Sister Norma.)

### III.—HOT BATHS.

The hot water cure is the one great remedy in Japan, no one having any belief in the virtues of cold water. Almost all baths are taken about 110 Fahr. The charm of the Japanese system of hot-bathing is proved by almost all foreign residents abandoning their cold tubs in its favour. There really seems to be something in the climate which renders hot baths more suitable than cold ones. Mr. Chamberlain says, "The Japanese passion for bathing leads all classes to make extensive use of hot mineral springs, in which their volcano-studded land abounds. Sometimes they carry their enjoyment of this natural luxury to an almost incredible extreme. At Kawanaka, a tiny spa, one of those places of which there are many in Japan, which look as if they were the very end of the world, so steep are the mountains shutting them in on every side, the bathers stay in the water for a month on end, with a stone on their laps to prevent them from floating in their sleep."

Your little handmaid will offer to aid you, and unless you prevent her will bring you numerous relays of refreshment during your enjoyment of this most luxurious bath. The Japanese have, with the exquisite cunning of their handiwork, devised such comfort and luxury in these baths, that even the Duke of Connaught was heard to exclaim that only to enjoy them was well worth a visit to Japan. It is quite customary for the mousmées to wait on both sexes whilst they are bathing; one of these little maids will take a tall Englishman off to his bath in the same way as we would a little child. When I went into my bath I used to place O Do San with her back to the outer door and threaten her with terrible things if she moved for one moment. These doors have no means of locking, and as the Japanese for politeness's sake never say "No" it is often difficult to know by the inflexion of the voice whether "Yes" is the negative or the affirmative.

The water which supplies these sulphur and iron baths in M'yanoshita comes two and a-half miles in bamboo pipes from the volcano of Ojigoku. There are also the same luxurious baths in which there is only a trace of salt and soda, which are used freely without medical advice, merely for pleasure's sake. Sulphur, iron, and salt are everywhere the chief minerals found in the Japanese springs; very few contain carbonic acid gas.

We were no sooner back in our rooms than the wily shampooer slowly felt her way into our room. Shampooing or massage, which has been a common cure for centuries in Japan, is a trade entirely in the hands of the blind, and who knowing Japan is not acquainted with the three melancholy notes of the blind shampooer's whistle and the thud, thud of his heavy bamboo on the pavements as he feels his way down the streets at night? There are always two or three attached to these great mountain hotels sitting in the corridors ready to capture the tired stranger on his return from the hot bath, which is always in readiness for every excursion party after a mountain climb to the beautiful Hakone Kake or Atami. If you do not forcibly resist the blind shampooer with very little ceremony, he or she commences operations at once, and though the sensation is not altogether a pleasant one it has much to recommend it to tired pedestrians. One mistake, however, the Japanese make is that they shampoo down, not upwards; a portion of the good done is thus neutralized, one object of scientific massage being to help back towards the centre, the blood which is lingering in the superficial veins.

After the evening meal a delicious languor becomes universal, and rocking chairs and hammocks are pulled out into the verandah. For those whose energies have not all been expended in the exertions of the day, there is the water-fall to be done by moonlight or a stroll through the primitive mountain valleys. And here I must say Sayonara to my memories of M'yanoshita, for with the gloaming I can hear the bull-frogs barking, and the grasshopper's crick, crick, and the weird twang, twang of the samisan as it comes up in the soft night from some paper home in the gorge below, and all the small memories crowd in which are profitable only to one who knows and loves the gentle land. For an absolute change from the prosaic everyday European life, probably no greater contrast could possibly be enjoyed than that which is secured in fair Japan.

### Worthing.

Hospital Saturday was duly honoured at Worthing this year, and the total amount collected was £36 13s. 3½d. Much credit should be given to the ladies who undertook the stalls and guarded the boxes patiently for so many hours. A grand parade of the town was also organised, and the procession contained representatives of every denomination, and a very gay display was made by the various societies and their bands.

### Where Was the Label?

A patient at Oldham Workhouse received a fatal dose of liquid atropia instead of whiskey, through the mistake of an over-worked nurse, who took the wrong bottle out of the cupboard in which drugs and medicines were kept. If the bottles were properly labelled such a blunder should be impossible, and in all cases it is better to have the stimulants kept in a special place of their own. Poisons should of course invariably be put into bottles of conspicuous form and colour, and stored in a cupboard by themselves.

### Notes and Queries.

### Queries.

(129) *Nursing Badges.*—Please tell me in The Hospital what can be used as a badge of nursing without interfering with any existing institution, medal or badge? Could a plain Maltese cross be used?—*Lucis.*

(130) *Male Nurses.*—Can you tell me where male attendants can be trained?—*H. N.*

(131) *Garrison Nursing Fund.*—I am anxious to find out about the "Garrison Nursing Fund," as I am anxious to get work as nurse in that capacity.—*T. S.*

(132) *Diabetic Food.*—Does common flour, after having the starch washed out by means of water being allowed to run over flour for a given time, become safe to give a diabetic patient in place of the gluten flour?—*E. G.*

(133) *The Trained Nurse.*—Where can this publication be obtained?—*Nemo.*

### Answers.

(129) *Nursing Badges (Lucis).*—Out of our power of giving a list of protected, but we imagine every form of the Maltese cross has been already appropriated. Meaningless badges worn without authority are not only not honourable but distinctly harmful to the wearer.

(130) *Male Nurses. (H. N.)*—There is no existing institution where male nurses are trained, but one is in process of completion in Germany. See our German Letter in last week's Mirror. For mental nursing, apply the Superintendent, Berrywood, Northampton.

(132) *Diabetic Food (E. G.)*—Gluten is the nitrogenous basis of flour. It is made by carefully washing flour in a stream of water. The resulting sticky mass is gluten with a very small percentage of starch adhering to it. A large proportion of gluten used for diabetic foods comes from the starch manufactories, where it is a waste product. The method described in the question is wasteful. The best way is to take the flour and wrap it up in butter cloth, 2½d. a yard, after the fashion of a meat pudding, and knead it well under a flow of water from a tap. Do this until a merely sticky mass remains, and the starch is washed away. It can be bought wholesale from the starch manufacturer.

(133) *The Trained Nurse (Nemo).*—This paper is published in America, and has no London office. We have forwarded your letter.

*Soldier's Nurse.*—Name and address must be given before we can answer querries.

☞ For The Position of the Workhouse Infirmary Nursing Association, Everybody's Opinion, &c., see page ccxxv. et seq.

### The Position of the Workhouse Infirmary Nursing Association.

In consequence of the recent action of the Bedford Board of Guardians in dismissing a nurse supplied to them by the W. I. N. Association, the latter has addressed a letter to the local press, in which the following paragraphs appear:—

"After several inconclusive letters from the clerk, who apparently wrote them without the authority of the Board, at the same time stating that the Guardians 'do not recognise the right of your Association to question them on such matters,' the guardians have at length given the following reply to our request for their reasons for dismissing Nurse Harcourt. Nurse Harcourt came to the Bedford Workhouse through the agency of the Workhouse Infirmary Nursing Association. After she had been at the workhouse a short time she expressed her distaste to certain portions of her duties. Before asking the Local Government Board to sanction her permanently the Visiting Committee of the Guardians saw Nurse Harcourt, when she admitted that she had so expressed herself, and also that she considered herself primarily the servant of that Association whose orders she would be bound to obey under penalties, in preference to any orders of the Guardians.

"These and other circumstances induced the committee to ask the Guardians whether they thought it desirable to apply to the Local Government Board to sanction Nurse Harcourt's appointment, whereupon the Guardians decided it was not desirable to do so.

"The Bedford Board of Guardians appear unable to recognise the position of our association as a charitable body working to ameliorate the sufferings of the sick poor in our workhouse infirmaries, and at the same time willing to help Guardians in obtaining the services of only well-trained nurses of thoroughly good character for their infirmaries.

"The Workhouse Infirmary Nursing Association was foundedin 1879 to promote the following objects: (1) To raise the standard of public opinion on the whole question of workhouse nursing; (2) to secure the appointment of trained ladies as matrons in all separate infirmaries; (3) to train and supply nurses to workhouse infirmaries in London and the provinces.

We would draw special attention to the last clause. We train our nures in recognised hospitals or infirmaries for one or two years at an average cost of £20, and this absorbs the greater part of our funds. But when the nurses are trained, how are we to secure their services specially for workhouse infirmaries? How do we endeavour to keep them out of the perhaps pleasanter paths of district, hospital, or private nursing? We make them sign an agreement that, in consideration of our paying their training fees, they undertake, 'For the next three years (at least) to take such posts as the committee will offer to them as day or night nurses in London or the country, to resign such if desired to do so by the committee, not to resign without permission, and to be considered as members of the Association.' The agreement further runs: 'If any nurse shall leave the Association during her training, or the three years following her training, she shall be obliged to repay to the Association the sum of £10.'

"In what way do we exercise our authority over our nurses? Undoubtedly to the advantage of the Guardians. Our nurses undertake not to resign an appointment without the sanction of the committee, and this rule is clearly in the interests of boards of guardians, since, through the influence of the Association and on account of these very rules, nurses are often persuaded to remain at a workhouse where the difficulties of the work would otherwise dispose them to leave soon after entering. It is obvious that this refers to the nurse wishing to leave for her own reasons. We could have no power if the Guardians asked her to resign for their own reasons. The secretary is constantly writing to tell a nurse that the committee cannot consent to her resigning until she has given the work a fair trial, and that they expect her to stay at least a year in a post, and so forth. We encourage nurses to stay on by medals, gratuities, &c. The correlative power to compel a nurse 'to resign if desired to do so by the committee' is one to which the Guardians are more likely to take exception, but a good Board will never have cause to experience its effect."

### A Case for Assistance.

A Nurse writes: Why do nurses who desire the pleasure and privilege of helping refrain because they can do only a little? If every nursing staff would produce an ordinary money-box for the purpose of receiving "mites," the sum needed would be speedily realised.

Sister Rose writes: I am sorry to see nurses have not come forward as they might to help a sister in distress. Maybe the reason is they think the little they can give will not go far, but if we all join surely the small sum could soon be raised. I, a nurse, suggest that all of us who read The Hospital should send sixpence. Most nurses could spare that small sum, and the money be quickly raised and a fellow worker helped.

*** We feel sure that the appeal we made for the nurse in distress has failed to meet the required response not from lack of interest and sympathy, but, as "A Nurse" points out, because nurses have hesitated to send such small sums as would have been numerous enough to secure the desired result. If institution nurses who sympathise would make a collection of small sums and send in the amount, that strikes us as the best method of effecting their object.

We are glad to be able to report that the readers of "The Mirror" are at last waking up to the urgency and claims of the case of the ex-Matron who needs but £20 to keep her little home together. This week we have to acknowledge the receipt of £4 4s., making the total receipts to date £10. It will be observed that the doctors, sisters, and nurses of the Birkenhead Hospital have combined to send £1 13s., and the nurses at Sheffield have collected amongst themselves 13s. 3d. Will not the matron or one of the nursing staff of a few other institutions kindly take the trouble to start a similar subscription, so that the remaining £10 may be raised during the current week? This is essentially a case of *bis dat qui cito dat*. Contributions should be sent as before to Miss Farrow or Miss Bell, Liverpool Homes for Aged Mariners, Egremont, Cheshire.

Received since August 20th: Dr. J. Bell, Edinburgh (second donation), 10s.; Miss B., Swansea, 10s,; Nurses, Sheffield, 13s. 3d.; Matron, Birkenhead Hospital (collected from doctors, sisters, and nurses), £1 13s.; Nurse F. A. H. (second donation), 6s.; Nurse G. (second donation), 2s.; without name, from Bournemouth, 5s.; S. Josephine (second donation), late of Mildmay, 2s. 9d.; total to date, £10.

### Minor Appointments.

The "Tongariro."—The New Zealand Shipping Company have engaged Miss Louisa Ellen Amor as stewardess on the "Tongariro," which has just started for a voyage round the world. Miss Amor was trained at the London Hospital and at the British Lying-in Hospital, Endell Street, and has done private nursing in Sydney, N.S.W., and held the post of stewardess on the Orient Line, on a voyage from Australia to London. We wish Miss Amor a prosperous voyage and a safe return.

Miss J. Ashby has been appointed assistant matron at Miss Duncan's Nursing Institution, Richmond.

### Wants and Workers.

Having broken my leg in nursing in a country parish, I shall be grateful if any one can lend or give a pair of spring crutches. Height, 5ft. 2in. Address Miss *Garrett, The Home, Knowbury, Ludlow.*

### Everybody's Opinion.

[Correspondence on all subjects is invited, but we cannot in any way be responsible for the opinions expressed by our correspondents. No communications can be entertained if the name and address of the correspondent is not given, or unless one side of the paper only be written on.]

### SHAM NURSES.

Dr. Garland writes: As your journal is the one chiefly devoted to the interests of the nurse, may I be allowed to offer a suggestion or two in its columns as to remedying the indiscriminate imitation of the nurses' costume. The dress is much adopted just now by almost all servant girls, to the evident satisfaction of their respective and thoughtless mistresses. Ladies surely could discourage this custom, as they have done the "fringe," by making it a *sine qu a non* on engaging a servant that such a dress will not be permitted. An appeal also to the matrons of small district hospitals and dispensaries should be made to prohibit any imitation uniform being worn by an attendant not a qualified nurse. A distinctive badge, either worked or in metal, might be issued by the respective training schools, or a universal badge (if preferred) might be worn either with or without a particular dress. I simply throw out these suggestions, of course leaving the details to be worked out by persons more competent to deal with such an important subject than I am.

### NURSING AT KIMBERLEY HOSPITAL.

Dr. Henry Draper Bishop writes from Glendalough, Weston-Super-Mare: Miss Ritchie's letter in The Hospital of the 25th inst. hardly touches the question of the nursing of Kimberley Hospital; it simply eulogizes the Matron, Sister Henrietta. Miss Ritchie states that I was appointed Junior House Surgeon. That is not so, as I would not take this post; I only filled it as a *locum tenens*. I resigned my appointment five weeks after obtaining it, for very good reasons, which I cannot now enter upon: and although I was only at Kimberley six months, with the knowledge I gained from the nurses of the hospital and my predecessor, Dr. Ashe, I can certainly form a good idea of the working of the nursing system. The alterations which the Warden sanctioned last year for the welfare of the nurses consisted of the substitution of an ordinary diet instead of a compulsory fish diet every Friday, as had been the case hitherto, and the delivery of nurses' letters to them as soon as they were received, instead of keeping them back for five hours (the old plan). These improvements, however, were granted, not willingly, but at the sole instigation of Dr. Ashe, who practically forced them upon the authorities. Surely no one could imagine that the nurses' dresses, which were made of any material that they fancied, were of one uniform thickness all the year round; the fact remains that the colour (black) is as unsuitable as could possibly be chosen, and that woollen things, if they wash at all, wash badly. As porters and wardmaids are not unknown in English hospitals, it did not seem necessary to state that we had such assistance at Kimberley; everyone would take it for granted that we had. From the whole of South Africa, Sister Henrietta is unable to find nurses sufficient for the needs of this, its premier hospital, simply because they will not stay in it longer than they are forced to; instead of a tribute to her administrative capacity, this is a grave reproach. The *Lancet* says that the Matron of this hospital is vested with powers which they venture to say are unexampled in respect to a similar official, and that this is deplorable and should not be permitted to continue; the *British Medical Journal*, says the system opens the door to much tyranny and abuse, and offers a premium to the worst evils of officialism. Most earnestly I urge nurses thinking of going to Kimberley to well weigh these statements in their minds before making a decision.

### For Reading to the Sick.

### HOME RULE.

### Motto.

He that ruleth his spirit is better than he that taketh a city. —*Proverbs of Solomon.*

### Verses.

Oh wean this self from me! that I
No more, but Christ, in me may live!
My vile affections crucify,
Nor let one hidden lust survive!
In all things, nothing may I see,
Nothing desire or seek, but Thee!

I keep under my body and bring it into subjection.—*St. Paul.*

Man, who man would be,
Must rule the empire of himself; in it
Must be supreme, establishing his throne
On vanquished Will, quelling the anarchy
Of hopes and fears. —*Shelley.*

Command thyself in chief. He life's war knows
Whom all his passions follow, as he goes.
—*George Herbert.*

### Reading.

We have heard a great deal lately in the political world about Home Rule; this is no place for politics . . . but I think we may connect these two words which we hear so often with some higher lesson, some spiritual thoughts, some consideration of our responsibilities, of our possibilities, of our dangers concerning Christian Home Rule. What sort of Home Rule is yours and mine? Could it be said of us as it was said of Abraham, who was called the "friend of God," "I know him, that he will teach his children and his household after him, and they shall keep the way of the Lord"? . . . Could you and I say with the "man after God's own heart," "I will walk within my house with a perfect heart"? . . . And if Christ came now to your home and mine, and lifted up His blessed hand, and said, "Peace be to this house," would He enter and find peace? No other rule is precious without this Home Rule, . . . the first great principle of which is self rule, self government, self command. . . . The first great duty we have to perform is to get a knowledge of self; . . . it was the conclusion of the old philosophers that the great work of man was to know himself; to know what was good in him, and what was bad, and to try to promote the good and expel the bad. See the contrast between God's rule and man's rule, between the converted and the unconverted heart. Have you ever noticed on the rail, in coming out of London or elsewhere, looking at the backs of the houses, and the small spaces apportioned to each, in one there was brightness and beauty and cultivated flowers, but in another were heaps of rubbish, dirt, and disorder? Or going into two houses you have seen the difference between the one where there was rule and cleanliness and order, the bright ornamentation that made the place cheerful and homelike, and the other where there was disorder—the home of the drunkard. It has been exhibited in pictures, but it is in reality far worse than any artist can paint. They saw there no rule, no God, no Christ, no conscience, no Holy Spirit, every man doing that which was right in his own eyes, regardless of his own happiness or that of his neighbours, and bringing misery to his home. But to him who ordered his conversation aright, doing his best, doing his duty, what light, what brightness, what warmth there was in his house; he had a foretaste in that home of heaven itself. . . . Let men make the heart right, let their life be cleansed by the inspiration of the Spirit, and the blood shed upon the Cross, and then they will have Home Rule, home happiness, and joy which will be continued through eternity.—*Dean Hole.*

### The Nightingale Fund.

REPORT FOR THE YEAR 1893.

The following is a statement of the probationer-nurses in the school at St. Thomas's Hospital during the year 1893: Remaining on January 1st, 39; admitted during the year, 60—99; resigned, or discharged as unsuitable for the work, 31; completed their year's training 33—64; remaining in the school on December 31st, 1893, of whom 16 were special or lady probationers, and 19 nurse probationers, 35.

Thirty-three probationer-nurses were entered on the register of nurses, after satisfactorily completing their year of training, and of these 15 were special probationers, and 18 nurse probationers. An unusual number of the probationers failed to complete the course after only a few weeks' trial, proving to be not strong enough for the duties required of them. Of the thirty-three probationers who completed the year's course in the home, all, with one exception, were taken on to the staff of St. Thomas's Hospital, and owing to the increased demand for supernumerary nurses, it is expected that in the future there will be sufficient vacancies on the staff for all the probationers who have become entitled to be placed on the register. This arrangement is equally advantageous to the hospital and to the school, and is preferable to sending probationers to take service during their second year in some other institution. One probationer was engaged by the Metropolitan and National Nursing Association, Bloomsbury Square, to be trained as a district nurse in connection with the Queen Victoria Jubilee Institute for Nurses.* From the opening of the school in June, 1860, to the end of 1893, a total of 1,156 candidates have been admitted, and 725 have after completing a year's training, received appointments in some public hospital or infirmary, or district nursing institution for the benefit of the sick poor.

The annual gratuity of £2 allowed by the regulations for the term of three years to the nurses who have completed a year's service satisfactorily in some approved hospital or other institution for the benefit of the sick poor, was awarded to 85 nurses, viz., 27 for their third year, 28 for their second year, and 30 for their first year.

Among the new appointments which former probationer-nurses who had completed their term of engagement to the Council have received during the year, may be mentioned the following, viz.: Elizabeth Jane Batchelor (admitted in 1879), Matron, Victoria Hospital, Hull; Mary Elizabeth Shipley (1880), Matron, Fulham Parochial Infirmary; Elizabeth Green (1884), Matron, Kent County Lunatic Asylum, Chartham; Margaret Ferguson (1887), Assistant Superintendent, Royal Infirmary, Liverpool; Mary G. A. MacDonell (1887), Matron, Cottage Hospital, Ashford; Emma Eliza Kelsall (1888), District Nurse, Paddington and Marylebone District Nursing Association; Margaret Ethel Howes (1888), Night Superintendent, St. Thomas's Hospital; Laura L. Ward (1888), Superintendent, Walworth District Nursing Association; Janet Eliza Rogers (1889), Ward Sister, Devon and Exeter Hospital; Alice Skelton (1889), and Eliza Gray Sidley (1889), both Head Nurses, Metropolitan Asylums Infectious Hospital, Tottenham; Jessie Main (1889), Matron, City Infectious Hospital, Liverpool; Ellen Bath (1889), Ward Sister, Devon and Exeter Hospital; Christian Stuart (1889), Assistant Superintendent, Parish Infirmary, Brownlow Hill, Liverpool; Louisa Papworth (1889), Ward Sister, Sussex County Hospital, Brighton. Others who had not yet completed their term were appointed as follows: One to be Theatre Sister, and four to be Ward Sisters, St. Thomas's Hospital; one, Ward Sister, Parochial Infirmary, Birmingham; one, Assistant Matron, Kent County Lunatic Asylum, Chartham; and one, District Nurse, Paddington and Marylebone District Nursing Association.

* Office at St. Katharine's Hospital, Regent's Park, London, N.W.

Miss Gordon, the matron of the hospital and superintendent of the school, reports that the probationers have worked well during the past year. The instruction of the probationers has been carried on as heretofore, as assistant nurses under the "sisters" in the hospital wards, by class teaching in the home and by lectures. Dr. Ord, the senior physician of the hospital, kindly gave six lectures. Dr. Sharkey's course consisted of eleven lectures, followed by an examination, and he also held quarterly examinations of those probationers who had completed nine months' training. Mr. Wyndham R. Dunstan, the lecturer on chemistry to the hospital, gave a course of twelve lectures on "The Chemistry of Air, Water, and Food," followed by an examination. Both examiners expressed themselves as much satisfied with the results of their examinations. From Miss Crossland, the "home" sister, the probationers have had the benefit, in addition to excellent instruction, of devoted care and attention to their personal comfort and moral well-being. And here it may be observed, quoting the words of Miss Nightingale, "that lectures, classes, and examinations, valuable as they are as instruments for teaching and testing technical knowledge and skill, are only a part of the essentials of nurse training. The personal moral qualities of the nurse, such as trustworthiness, patience, a firm kindness, sound judgment and a wise devotion to her work for the work's sake, all qualities of the head and heart, are the absolutely necessary complement of the technical part. These qualities may be taught by moral training and discipline, and may be tested by results, but can be taught by no lectures, tested by no examinations, and recorded by no public register." The committee believe that such teaching has not been neglected in this school, and they desire to thank those who, by current supervision, example and sympathy, have so carried out the training.

The thanks of the committee are due to Dr. Ord, Dr. Sharkey, and Mr. Dunstan for their valuable assistance, and also to Dr. Turner and Mr. Stabb, the resident medical officers, as well as to Mr. Clutton and Dr. Ballance, for kind services rendered to the probationers during illness. Dr. Sharkey reports that the health of the probationers during the year had been good.

Miss E. Vincent, the matron of St. Marylebone Parochial Infirmary, Notting Hill, reports that there were 12 probationers in the training school on January 1st, 1893, and 20 were admitted during the year. Of these 11 completed their year's probation, and were then taken on the staff of the infirmary; 7 left, being from various causes unsuitable, leaving 14 remaining in the school on December 31st. Former probationer-nurses, who have completed their term of engagement to the Council as parish infirmary nurses, received new appointments during the year as follows: Two, Head Nurses at St. Marylebone Infirmary; four, Extra Nurses on the staff of the Royal Infirmary, Edinburgh; one Head Nurse and one Night Superintendent at St. Saviour's, Southwark, Parochial Infirmary; Head Nurse, Mill Road Parochial Infirmary, Liverpool; Night Superintendent, Royal Hospital, Belfast; Night Superintendent, Hospital for Consumption, Brompton; District Matron, Limehouse East London District Nursing Association; Nurse, Paddington and Marylebone District Nursing Association; two to be trained as District Nurses for service under the Queen Victoria Jubilee Institute for Nurses; and Nurse-Matron, Cottage Hospital, Woking.

Eight nurses received the gratuity for their first year of service, and ten for their second year of service as parochial infirmary nurses. Mr. Lunn's lectures and Miss Moriarty's classes were regularly attended by the probationers. To them and to Miss Vincent the thanks of the Council are due.

The able Secretary is Mr. H. Bonham Carter, 5, Hyde Park Square, W.

